# Nano-Encapsulated Berberine Is a Potential Therapeutic Agent for Adipose Tissue Browning in C57BL/6J Mice

**DOI:** 10.3390/medicina61101738

**Published:** 2025-09-24

**Authors:** Aslıhan Alpaslan, Kübra Uçar Baş, Elif Didem Örs Demet, Dilem Tuğal Aslan, Tuba Reçber, Süleyman Can Öztürk, Tugba Gulsun, Mustafa Çelebier, Zeynep Göktaş

**Affiliations:** 1 Department of Nutrition and Dietetics, Faculty of Health Sciences, Hacettepe University, 06100 Ankara, Türkiye; aslihan.alpaslan11@hacettepe.edu.tr (A.A.); kubraucar@hacettepe.edu.tr (K.U.B.); elif_didem@hotmail.it (E.D.Ö.D.); dilemtugal@hacettepe.edu.tr (D.T.A.); 2 Department of Nutrition and Dietetics, Faculty of Health Sciences, Akdeniz University, 07058 Antalya, Türkiye; 3 Department of Nutrition and Dietetics, Faculty of Health Sciences, Osmaniye Korkut Ata University, 80600 Osmaniye, Türkiye; 4 Department of Nutrition and Dietetics, Faculty of Health Sciences, Necmettin Erbakan University, 42090 Konya, Türkiye; 5 Department of Analytical Chemistry, Faculty of Pharmacy, Hacettepe University, 06100 Ankara, Türkiye; tuba.recber@hacettepe.edu.tr (T.R.); celebier@hacettepe.edu.tr (M.Ç.); 6 Laboratory Animals Research and Application Center, Hacettepe University, 06100 Ankara, Türkiye; scanozturk@hacettepe.edu.tr; 7 Department of Pharmaceutical Technology, Faculty of Pharmacy, Hacettepe University, 06100 Ankara, Türkiye; tgulsun@hacettepe.edu.tr

**Keywords:** berberine, nano-encapsulation, adipose tissue, browning, obesity

## Abstract

*Background and Objectives:* Berberine is a promising phytochemical for obesity prevention due to its effects on adipogenesis and adipose tissue browning. Despite the benefits shown in cell studies, the clinical use of berberine is limited because of its low stability and bioavailability. *Materials and Methods*: Our study aimed to investigate the effects of intravenous liposomal and free berberine on body weight and adipose tissue browning in C57BL/6J mice. The mice were divided into two main groups for obesity prevention and treatment: the prevention group received treatment with a high-fat diet for 10 weeks; the recovery group received treatment after 10 weeks on a high-fat diet. Treatments included liposomal berberine (10 mcM), free berberine (10 mcM), and void nano-encapsule, and PBS was used as a control. *Results:* Berberine did not affect body weight in the prevention group. In the treatment group, nano-berberine reduced weight gain, while free berberine caused weight loss (*p* < 0.05). PRDM16 and CIDEA expressions in white and brown adipose tissues were higher in the berberine-treated groups (*p* < 0.05). No changes were observed in UCP1, PGC1α, C/EBPβ, and FABP4 expressions. The protein concentrations of UCP1, PGC1α, and PPARγ did not change. *Conclusions:* The effects of liposomal berberine on gene expression and protein concentrations were not different from the free form, but the nano form had higher stability.

## 1. Introduction

Obesity affects many systems, leading to various health issues such as cardiovascular diseases, metabolic syndrome, type 2 diabetes, and some types of cancer [1,2,3]. According to the World Health Organization, in 2016, 39% and 13% of adults worldwide were overweight and obese, respectively [4]. In 2020, 39 million children under the age of 5 were overweight or obese. Obesity is considered a 21st-century epidemic due to its high prevalence [5]. Since the main cause of obesity is positive energy balance, treatment methods focus on reducing energy intake and/or increasing energy expenditure [6].

Body fat mass consists of white and brown adipose tissues [7]. In these tissues, white and brown adipocytes are predominantly found, respectively [8]; however, a new form of adipocyte called beige/brite has been discovered in the white adipose tissue [9]. Beige adipocytes, under appropriate stimuli, function similarly to brown adipocytes. The primary characteristic of brown adipocytes is their ability to induce non-shivering thermogenesis through high mitochondria content and Uncoupling Protein 1 (UCP1). Thermogenesis, a component of energy expenditure, is the physiological process by which the body generates heat [9]. Increasing thermogenesis can increase energy expenditure, making it important in preventing obesity. Berberine (BBR) is a natural isoquinoline alkaloid and polyphenol extract widely used in traditional medicine, especially in China and many other Asian countries [10]. It is believed to have many positive effects on health, including anti-obesity, hypoglycemic, hypolipidemic, hypotensive, and anti-inflammatory effects [10,11,12,13]. Studies have shown that one mechanism of BBR’s anti-obesity effect is related to adipose tissue browning [9,14]. Despite its positive health effects, BBR has low stability and bioavailability [15]. Various methods have been tested to optimize the structure of phytochemicals like BBR [16,17,18]. One of these methods is lipid-based nano-encapsulation, which is believed to increase BBR’s stability, create a biocompatible and biodegradable capsule, and thereby enhance BBR’s effectiveness [16].

In this study, we aimed to investigate the effects of intravenous administration of nano-encapsulated and free BBR on body weight, brown adipose tissue (BAT) activation, and white adipose tissue (WAT) browning in female C57BL/6J mice.

## 2. Materials and Methods

### 2.1. Materials

Berberine (purity ≥ 95%) was purchased from Cayman Chem (10006427, Ann Arbor, MI, USA). L-α-Phosphatidylcholine (soy PC) and Cholesterol (purity ≥ 99%) were purchased from Sigma-Aldrich (P5638 and C8667, St. Louis, MO, USA). A syringe filter was purchased from Alwsci Technologies (SF13-PVDF-22LP, Shaoxing, China). A sterile syringe filter was purchased from ISOLAB Laborgeräte GmbH (094.07.009, Eschau, Germany). One Step-RNA Reagent, used for total ribonucleic acid (RNA) isolation, was purchased from Bio Basic (BS410A, Markham, ON, Canada). cDNA Synthesis Kit (G236) and BlasTaq™ 2X qPCR MasterMix (G891) were purchased from Applied Biological Materials Inc-ABM (Vancouver, BC, Canada). Enzyme-linked immunosorbent assay (ELISA) kits for UCP1 (SEF557Mu), peroxisome proliferator-activated receptor γ (PPARγ) (SEA886Mu), and PPARγ-co-activator-1α (PGC1α) (SEH337Mu) were purchased from USCN (Wuhan, China).

### 2.2. Animals

This study was conducted with the permission of the Hacettepe University Animal Experimentations Local Ethics Board (Decision No: 2020/04-05). Forty-eight female C57BL/6J mice, aged 6–8 weeks and from the same lineage, were obtained from the Hacettepe University Laboratory Animals Research and Application Centre (HUDHAM). The interventions, euthanasia, and subsequent surgical procedures of the mice were carried out at HUDHAM. The mice were maintained under standard environmental conditions with a temperature of 22 ± 2 °C, a 12-h light/dark cycle, and 45% humidity. To standardize conditions, the mice were fed a chow diet (4 kcal/body weight) for the first week. After standardization, the mice were divided into 8 groups and placed in different cages. Throughout the study, the mice had water and feed ad libitum.

After standardization, the mice were randomly divided into 8 groups, half in prevention groups and the other half in recovery groups. Prevention groups were treated with a high-fat diet (60% of energy from fat) along with treatment for 10 weeks. Recovery groups were fed a high-fat diet for 10 weeks, followed by 10 weeks of treatment with the high-fat diet. These groups were further divided into four subgroups: nano-BBR (10 mcM), free BBR (10 mcM), void, and control. The dose of free and nano-BBR was selected based on preliminary in vitro experiments in 3T3-L1 preadipocytes, where this concentration showed the most pronounced browning effect. Treatments were administered intravenously via tail vein injection at a volume of 100 µL per mouse, once weekly. At the end of the interventions, blood samples were taken from the mice under intraperitoneal ketamine/xylazine anesthesia via intracardiac puncture, followed by euthanasia via cervical dislocation. The inguinal WAT and BAT were dissected from each mouse.

### 2.3. Treatment Preparation and Characterization

Nano-BBR, free BBR, and void were prepared as indicated in the Supplementary Materials. The physical properties of nano-BBR and void, such as particle size, zeta potential, and polydispersity index (PI), were visualized using a dynamic light scattering device. Nano-BBR morphology was visualized using a scanning electron microscope (SEM). The chemical properties were determined using high-performance liquid chromatography (HPLC), and the encapsulation efficiency (EE) and loading capacity (LC) were calculated using the following formulation:

EE% = 100 × (Amount of active substance − Amount of free active substance)/Amount of active substance

LC% = 100 × (Amount of active substance − Amount of free active substance)/Nanoparticle weight

### 2.4. Nano-BBR Stability

Free and nano-BBRs were examined in transparent and black tubes for 5 days at three different temperatures (4 °C, 22 °C, and 37 °C). To evaluate physical stability, the average particle size, zeta potential, and PI value of nano-BBR were measured every 2 h for the first 10 h, every 24 h for 5 days, and on the 10th day. Chemical stability of free and nano-BBR was evaluated by HPLC. Chromatographic separations were performed on C18 column (75 × 4.6 mm, 3 µm) using an isocratic mobile phase of acetonitrile and 0.1% formic acid–water (40:60, *v*/*v*). Chemical stability was measured daily for 5 days and on day 10, and results were expressed the percentage of BBR remaining relative to baseline.

### 2.5. Tissue Homogenization, RNA Extraction, and Real-Time PCR

The collected inguinal WAT and BAT were homogenized in One Step-RNA Reagent using a Bioprep-6 homogenizer with 9 iron beads. Total RNA was isolated from inguinal WAT and BAT for real-time polymerase chain reaction (PCR) analysis using One Step-RNA Reagent according to the protocol. The obtained RNA was reverse-transcribed to complementary DNA (cDNA) using a cDNA Synthesis Kit. Real-time PCR was performed using the LightCycler^®^ 480 (Roche, Basel, Switzerland) instrument according to the protocol of the BlasTaq™ 2X qPCR MasterMix Kit. β-actin was used as the housekeeping gene for CCAAT/enhancer-binding protein β (C/EBPβ), PPARγ, PGC1α, and cell death-inducing DNA fragmentation factor-like effector A (CIDEA), fatty acid-binding protein 4 (FABP4), and 36B4 was used as the housekeeping gene for UCP1 and PR domain containing 16 (PRDM16). Primer sequences of target genes are listed in the Supplementary Table S1. The data were analyzed using the 2^−ΔΔ^ Ct method and presented as expression fold changes compared to the control group.

### 2.6. Protein Extraction and ELISA

Total protein was isolated from serum, inguinal WAT, and BAT using One Step-RNA Reagent according to the protocol. The UCP1, PPARγ, and PGC1α protein levels in serum and homogenized inguinal WAT and BAT were measured with ELISA kits (SEF557Mu, SEA886Mu, SEH337Mu) according to the manufacturer’s instruction manual.

### 2.7. Statistical Analysis

Data analysis was performed using SPSS 22.0. Distribution normality was checked using skewness and kurtosis tests. ANOVA was used to test the significance of differences between the means of three or more independent groups. Data are presented as the means ± standard error of the mean (SEM). Differences were considered statistically significant when the *p*-value was <0.05.

## 3. Results

### 3.1. Nano-BBR Exhibited a More Sustained Release Behavior and Stability Compared to the Free Form

The characteristic properties of free and nano-BBRs have been provided in the Supplementary Materials. According to SEM imaging, nano-BBR had a spherical structure with a particle size of 207 nm. The *p* value was measured as 0.3, and the zeta potential was determined as −27 mV. According to the formulations, the encapsulation efficiency and loading capacity values of nano-BBR were found to be 96% and 21.1%, respectively.

The in vitro release study was conducted in a dark environment to prevent BBR degradation. The amount of BBR released from free BBR decreased gradually after 4 h, while the release of BBR from nano-BBR continued for 24 h. The in vitro release profile is provided in Supplementary Table S4.

When physical stability was tested, it was observed that nano-BBR maintained its particle size, PI value, and zeta potential at 4 °C in darkness. After 10 days, the chemical stability of free BBR decreased under light conditions, but when stored in darkness for 10 days, it degraded to a lesser extent. On the other hand, nano-BBR maintained its chemical stability both in darkness and light. When evaluated at different temperatures, chemical stability at 4 °C and 22 °C was similar. The nano-encapsulation of BBR enhanced its chemical stability against light and room temperature. The detailed results of the stability tests are provided in Supplementary Tables S2 and S3.

### 3.2. BBR Treatment Provided Weight Control in the Recovery Group

As shown in Table 1, the weight changes in mice receiving different treatments in the prevention group were similar. Mice receiving free BBR in the recovery group lost weight after treatment, and those receiving nano-BBR had the least weight gain. There is a difference of 2.8 ± 1.53 g between the weight gain in the void group and the weight loss in the free BBR group (*p* = 0.042).

### 3.3. Gene Expression and Protein Levels of Browning Markers

**Figure 1:**
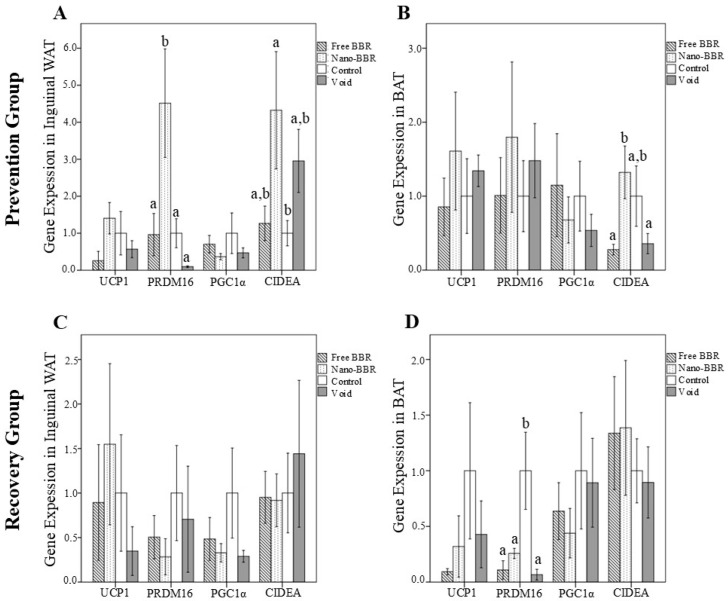
The gene expression levels of browning markers in inguinal white and brown adipose tissues of mice in the prevention (**A**,**B**) and recovery (**C**,**D**) groups. Mice were divided into four subgroups: nano-BBR (10 µM, i.v., weekly), free BBR (10 µM, i.v., weekly), void and control (PBS). Data are presented as mean ± SEM (*n* = 6 per group). One-way ANOVA followed by LSD post hoc test was applied. Bars not sharing the same letter are significantly different at *p* < 0.05. Abbreviations: BAT, brown adipose tissue; BBR, berberine; CIDEA, cell death-inducing DNA fragmentation factor-like effector A; PGC1α, PPARγ-co-activator-1α; PRDM16, PR domain containing 16; UCP1, uncoupling protein 1; WAT, white adipose tissue.

We investigated the effect of different treatments on browning markers in inguinal WAT (Figure 1A) and BAT (Figure 1B) of mice in the prevention group. After interventions, PRDM16 expression in the nano-BBR group was higher in both inguinal WAT and BAT than the free BBR, control, and void groups. This difference was statistically significant in inguinal WAT (*p* = 0.012, *p* = 0.013, *p* = 0.003, respectively). CIDEA expression in the inguinal WAT of mice receiving nano-BBR was higher than that in the control group (*p* = 0.029). Additionally, CIDEA expression in their BAT was higher than in the free BBR and void groups (*p* = 0.017, *p* = 0.021, respectively). UCP1 expression in both inguinal WAT and BAT of the nano-BBR group was higher than in the other groups, although this difference was not statistically significant. There was no significant difference in the expression levels of PGC1α in tissues among the treatment groups (*p* > 0.05).

Figure 1C,D present the effects of interventions on the browning markers in the inguinal WAT and BAT of mice in the recovery group, respectively. In the inguinal WAT, the expression of browning markers was not different among the treatment groups. However, in the inguinal WAT of the nano-BBR group, UCP1 expression was higher than other groups, but this difference was not statistically significant (*p* > 0.05). In the BAT of the control group, PRDM16 expression was higher than other groups (*p* < 0.05). The expression of other browning markers in BAT did not differ among the groups (*p* > 0.05).

Table 2 presents the protein levels of the browning markers UCP1 and PGC1α in inguinal WAT, BAT, and serum. There was no difference in the protein levels of these markers among mice in the prevention group receiving different treatments. When the recovery group was evaluated, the serum protein levels of UCP1 and PGC1α in mice that received void treatments were higher than other groups (*p* < 0.05). The protein level of PGC1α in the inguinal WAT of control mice was higher than other groups (*p* < 0.05).

### 3.4. Gene Expression and Protein Levels of Adipogenesis Markers

**Figure 2:**
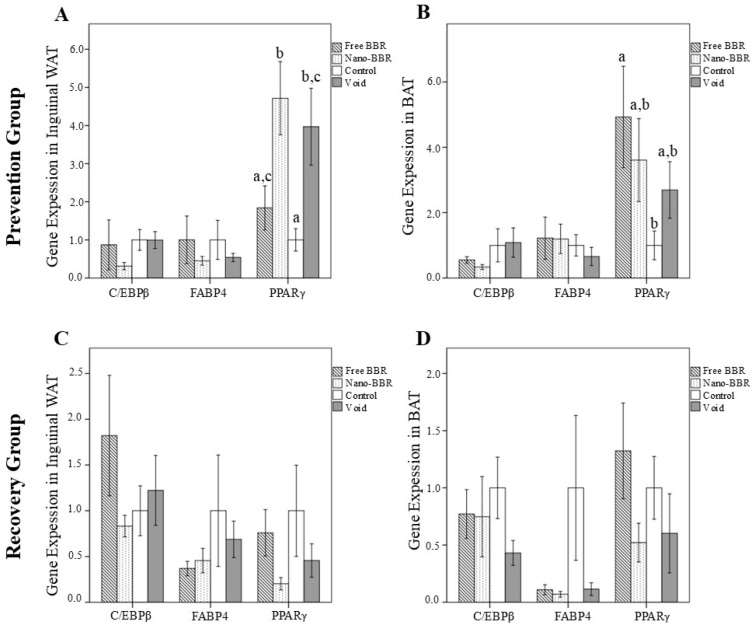
The gene expression levels of adipogenesis markers in inguinal white and brown adipose tissues of mice in the prevention (**A**,**B**) and recovery (**C**,**D**) groups. Mice were divided into four subgroups: nano-BBR (10 µM, i.v., weekly), free BBR (10 µM, i.v., weekly), void and control (PBS). Data are presented as mean ± SEM (*n* = 6 per group). One-way ANOVA followed by LSD post hoc test was applied. Bars not sharing the same letter are significantly different at *p* < 0.05. Abbreviations: BAT, brown adipose tissue; BBR, berberine; C/EBP-β, CCAAT/enhancer-binding protein beta; FABP4, fatty acid-binding protein 4; PPARγ, peroxisome proliferator-activated receptor-γ; WAT, white adipose tissue.

We investigated the effect of different treatments on adipogenesis markers in inguinal WAT (Figure 2A) and BAT (Figure 2B) of mice in the prevention group. Although there was no difference in the mice’s BAT, the gene expression of C/EBPβ and FABP4 in the inguinal WAT of the nano-BBR group was lower compared to other groups (*p* > 0.05). PPARγ expression in the inguinal WAT of the nano-BBR group was higher compared to the free BBR and control groups (*p* = 0.024 and *p* = 0.005, respectively). In the BAT, PPARγ expression in the free BBR group was higher compared to the control group (*p* = 0.029).

The expression of adipogenesis markers in the inguinal WAT and BAT of the recovery group, as shown in Figure 2C,D, did not differ among the groups.

Table 2 presents the levels of the adipogenesis marker PPARγ in inguinal WAT, BAT, and serum. PPARγ levels in the BAT and serum of the prevention group receiving free BBR were low, as were the levels in the inguinal WAT and serum of the recovery group receiving free BBR (*p* > 0.05). Additionally, the PPARγ level in the BAT of the recovery group receiving nano-BBR was also lower than in other treatment groups (*p* > 0.05).

## 4. Discussion

Phytochemicals show promise for obesity prevention and treatment, but their low bioavailability limits their use. Nano-technological methods seem to be the primary solution to overcome this issue. Having a suitable structure for the nanoparticle ensures better functionality. Many methods have been developed for BBR nano-encapsulation; in this study, we chose the liposome method due to its advantageous features, such as similarity to the cell membrane structure, impact on BBR stability, biodegradability, low toxicity, and relatively easier and faster production [19]. Particle size, zeta potential, PI value, physical and chemical stability, encapsulation efficiency, and loading capacity are used to determine the characteristics of nanoparticles [20]. The size of a nano-encapsulated phytochemical can affect its therapeutic properties [21]. Factors such as the half-life of the treatment, absorption, distribution, and cellular uptake can be influenced by size. In this study, we determined the nanoparticle size using both dynamic light scattering and SEM. The spherical-shaped nano-BBR had a size of 207 nm. Although the ideal size for nano-BBR is not precisely defined, in general, nanoparticles smaller than 10 nm may not escape the renal filtration barrier [21]. On the other hand, nanoparticles larger than 200 nm may be removed from circulation by the complement system and accumulate in the liver and spleen. Zeta potential determines the surface charge of the nanoparticle, which in turn affects its pharmacokinetics [20]. We measured the zeta potential value of nano-BBR as −27 mV. The reason for the negative zeta potential was soy PC, and this value indicates that the particles are stably dispersed [20]. Low PI values indicate that the particle size is homogeneous, while values greater than 0.3 indicate heterogeneity [20]. In this study, we measured the PI value of nano-BBR as 0.3. In our study, we calculated that 96% of the BBR molecules were successfully encapsulated. The encapsulation efficiency of the liposomal nano-BBR we prepared is higher than the nano-BBR prepared using the thin-film hydration method (81%) [22]. In a study investigating the effect of nano-BBR on tumor growth, the effects of thin film hydration, reverse phase evaporation, extrusion, high-pressure homogenization, gel filtration, and dialysis methods were compared [23]. The encapsulation efficiency of liposomal BBR prepared via thin film hydration, extrusion, and gel filtration methods containing 5 mol% polyethylene glycol (14%) was found to be higher than other methods. When compared with other data in the literature, it is seen that our formulation is effective. We calculated the loading capacity of the nano-BBR we prepared as 21.1%. High encapsulation efficiency and loading capacity reduce the amount of nanocarriers required and thereby decrease potential side effects. Although the acute effects of nanoparticles are evaluated in toxicity and side effect studies, the issues that may arise with long-term intake and accumulation in the body are not clear [20].

In the in vitro study, free BBR showed a sharp decline in release after 4 h, whereas nano-BBR continued to release steadily for 24 h, indicating a burst release for the free form and a sustained release for the nano-form. We observed that both free and nano-BBRs maintained their physical and chemical stability at around 4 °C and in a dark environment for approximately 10 days. The differences in baseline profiles reflected their distinct degradation patterns: free BBR showed burst release and rapid degradation, particularly under light exposure, while nano-BBR remained more stable due to protection within the liposomal vesicles. Nano-BBR, both in light/dark conditions and under different temperature settings, consistently remained more stable compared to the free form. The liposomal structure protected nano-BBR from oxidation and leakage of BBR. Berberine’s nano-encapsulation provides better protection against factors such as light and high temperatures, which affect its stability. The successful physical and chemical stability of nano-BBR may be due to the cholesterol it contains. Nguyen et al.’s in vitro and in vivo studies showed that chitosan-coated nano-liposomal BBR remained more stable than uncoated ones at both 4 °C and 25 °C over 30 days [24]. Additionally, increasing the chitosan ratio (0.1% to 0.3%) positively impacted stability. In the same study, the release behavior was also investigated, revealing that chitosan-coated BBR was released more slowly than the uncoated form. The liposomal layer can be increased to further enhance BBR stability. Previous studies with chitosan-coated liposomes, micelles, and other liposomal formulations reported improved stability, bioavailability, and biological activity of BBR [22,23,24]. In comparison, our liposomal nano-BBR showed high stability but did not markedly enhance gene or protein expression compared to free BBR. Most nano-BBR studies focus on stability and bioavailability but rarely examine browning or adipogenesis biomarkers.

Berberine has preventive and therapeutic effects against health issues such as obesity and comorbidities like type 2 diabetes, cardiovascular diseases, and cancer [25]. The underlying cause of these effects is thought to be BBR’s promotion of various mechanisms, such as inhibiting adipogenesis and stimulating adipose tissue browning [26,27,28]. In the 10-week obesity prevention, we did not observe a significant effect of either free BBR or nano-BBR on the body weight of the mice. In the recovery group, mice were administered nano-BBR once a week for 10 weeks, which reduced weight gain. Interestingly, mice receiving free BBR lost weight. In the study by Zhang et al., administering 50 and 100 mg/kg of BBR daily for 30 days reduced body weight in both diabetic rats and the control group [29]. Additionally, BBR reduced fasting blood glucose and suppressed hepatic gluconeogenesis. Another study showed that administering 150 mg/kg of BBR orally daily for 8 months to rats on a high-fat diet reduced weight gain and lipolysis [30]. In a study examining the effect of BBR on obesity prevention, rats fed both chow and high-fat diets were administered 100 mg/kg of BBR orally daily for 18 months [31]. In both groups, the weight gain was less than those not receiving BBR. Moreover, the group receiving BBR along with the high-fat diet had a weight similar to those on the chow diet throughout the study. Similar studies demonstrating that BBR reduces weight gain or even induces weight loss typically use doses in the range of 100–200 mg/kg [25]. Treatments were often administered daily, and the treatment duration was longer than our study. When comparing the effects of obesity prevention and recovery, the therapeutic effect of BBR appears to be more prominent in high-fat diet-fed, diabetes-induced animal studies. In our study, the change in body weight in the groups receiving BBR in the recovery group, although insufficient to draw a definitive conclusion, is a clinically favorable finding.

With the advancement of positron emission tomography (PET) combined with X-ray computed tomography (CT) imaging techniques, the presence of BAT has been observed in adult humans. Identifying factors that stimulate brown adipose tissue is critical for combating the obesity epidemic as these factors increase energy expenditure. Some dietary factors, such as BBR, are among these factors. The mechanisms underlying the effects of BBR on obesity and its comorbidities are still being investigated, and the number of studies in this intriguing field is increasing day by day. The most emphasized mechanism today is the ability of BBR to increase the expression of brown adipose tissue markers such as UCP1, PRDM16, PGC1α, and CIDEA, while reducing the expression of adipogenesis markers such as C/EBPβ and FABP4 [9]. Although PPARγ is considered a marker of adipogenesis, it has also been shown to activate brown adipocyte-specific genes [32]. Xu et al. showed that administering 25 and 100 mg/kg of oral BBR to C57BL/6J mice for 12 weeks increased UCP1 expression [32]. They also concluded that BBR activates the adenosine monophosphate-activated protein kinase/Sirtuin 1 (AMPK/SIRT1) pathway, which is a key regulator of energy metabolism and mitochondrial biogenesis. This activation increases the acetylation of PPARγ, consequently enhancing the expression of the thermogenic gene UCP1. According to Cai et al., while BBR has beneficial effects on lipid and glucose metabolism, these effects are not always accompanied by changes in the expression of expected markers like UCP1 [33]. In the prevention group, we found high UCP1 gene expression in both inguinal WAT and BAT of mice receiving nano-BBR (*p* > 0.05). As expected, the non-significant increase in expression did not impact the weight change in this group of mice. Additionally, in our study, BBR treatment did not affect the protein concentration of UCP1 in serum, WAT, and BAT. Factors such as the dose of BBR, duration and frequency of treatment, and the conditions under which the intervention is conducted can influence expression levels. PGC1α plays a critical role in the browning effect of BBR. Zhang et al. emphasized that PGC1α activation is necessary for UCP1 expression, indicating that PGC1α exerts its effects not only on its own but also by inducing UCP1 [28]. PGC1α is generally expected to be more expressed in BAT compared to WAT [34]. In our study, we did not observe a significant difference in PGC1α expression among treatments. However, as expected, the expression in BAT was higher compared to that in WAT. The protein concentration of PGC1α in serum, WAT, and BAT was not as expected among the treatment groups. PRDM16 is one of the markers that regulate the functions of brown and beige adipocytes [35]. This gene influences highly important processes such as the expression of other browning genes and mitochondrial biogenesis. When highly expressed, PRDM16 binds to the promoter region of UCP1, leading to upregulated UCP1 [36]. Additionally, PRDM16 interacts with factors such as PPARγ and PGC1α, enhancing their transcriptional activities. We found higher PRDM16 expression in both WAT and BAT of mice in the prevention group receiving nano-BBR compared to the other groups. This is an expected outcome, but there was no difference between treatments in the recovery group. PRDM16 has different expression levels in various fat depots. Its expression in BAT is expected to be higher than WAT. We did not find any differences in PRDM16 expression between BAT and WAT. Both Wu and Hirai, in their respective studies, reported that BBR treatment increased CIDEA expression [37,38]. In the study by Wu et al., BBR treatment administered daily at a dose of 1.5 mg/kg for 6 weeks to obese db/db mice increased the mRNA levels of other browning factors, such as UCP1 and PGC1α, in addition to CIDEA. Hirai et al. also found similar results with a daily treatment of 5 mg/kg BBR. We also observed that CIDEA expression in both WAT and BAT of mice in the prevention group receiving nano-BBR was higher than in the control and void groups, respectively. Nano-BBR affected PRDM16 and PPARγ expressions in WAT, as well as CIDEA expression in both WAT and BAT, more than free BBR. However, the protein concentrations between these two groups were not significantly different. This discrepancy may be due to post-transcriptional regulation, differences in the timing of gene versus protein responses, or the limited dosing regimen used. Future studies should therefore evaluate longer intervention periods and more frequent dosing to better capture the dynamics between mRNA and protein changes.

Berberine suppresses 3T3-L1 adipocyte differentiation while reducing the transcriptional activity and protein levels of C/EBPβ. Zhang et al. treated mice with 250 mg/kg of berberine daily for 4 weeks, and C/EBPβ expression, which plays an important role in adipogenesis, was downregulated [39]. In the 3T3-L1 cell study, treatment with BBR in the range of 0–40 mcM downregulated several adipogenesis markers, including C/EBPβ and FABP4 [40]. In this study, we did not observe any effect of BBR on C/EBPβ and FABP4 expressions in either the prevention or recovery group. PPARγ is an important marker in both white and brown adipocyte development [41]. Therefore, it cannot be classified as clearly as the other markers in this study. Although there is more evidence suggesting that it is a marker of adipogenesis, it is also known that PPARγ plays an important role in UCP1 stimulation, which is a fundamental marker of browning [32,41]. We found higher PPARγ expression in the WAT and BAT of mice receiving BBR treatment in the prevention group.

This study has some limitations that should be acknowledged. First, only female C57BL/6J mice were used; therefore, the findings may not be directly generalizable to both sexes or to other strains. Second, the intervention period and dosing frequency were limited. Intravenous injections were administered once weekly to minimize stress to the animals and to maintain a practical experimental design; however, given the short half-life of berberine, this regimen may have contributed to the modest and sometimes inconsistent biological effects observed. Third, we did not investigate beige adipocyte-specific markers but instead focused on general browning- and adipogenesis-related markers. Finally, systemic metabolic parameters such as glucose tolerance, lipid profiles, or energy expenditure were not assessed. Including these functional outcomes in future studies will be important to establish a clearer link between nano-BBR-induced molecular changes in adipose tissue and overall metabolic health. These limitations should be addressed in future studies to more comprehensively evaluate the biological effects of nano-BBR.

## 5. Conclusions

In this study, the effects of liposomal berberine on gene and protein expression were not markedly different from the free form. However, nano-encapsulation improved its stability and storage conditions, making it a promising approach for preserving berberine. While gene expression markers showed favorable changes, the corresponding protein-level findings were not consistent, indicating that these results should be interpreted with caution. Moreover, the once-weekly intravenous dosing regimen applied here may not fully capture the pharmacokinetic profile of berberine, which could influence the translational relevance of our findings. Future studies exploring higher doses, more frequent administrations, longer treatment periods, or clinically relevant routes may provide further insights into the physiological effects and therapeutic potential of nano-encapsulated berberine.

## Figures and Tables

**Table 1:** Pre- and post-intervention body weights and weight differences in the prevention and recovery groups ^†, ‡, §^.

		Before Intervention (g)	After Intervention (g)	Body Weight Changes (g)
Prevention group	Free BBR	19.2 ± 1.56	24.5 ± 2.45	4.8 ± 1.88
	Nano-BBR	18.3 ± 1.15	24.0 ± 1.64	5.7 ± 0.79
	Void	18.6 ± 1.02	23.1 ± 2.07	4.5 ± 1.93
	Control	18.5 ± 1.56	22.4 ± 1.80	3.9 ± 2.04
	*p* = 0.325			
Recovery group	Free BBR	25.7 ± 2.25	24.5 ± 1.81	−1.2 ± 1.40 ^a^
	Nano-BBR	24.0 ± 2.47	24.3 ± 0.85	0.4 ± 2.16
	Void	22.6 ± 1.28	24.3 ± 1.10	1.6 ± 0.33 ^b^
	Control	24.4 ± 1.83	24.9 ± 1.36	0.5 ± 1.63
	*p* = 0.042			

^†^ Data were analyzed by one-way ANOVA. ^‡^ Data are presented as mean ± SD. ^§^ Means within a column without a common letter differ, *p* < 0.05. Abbreviations: BBR, berberine.

**Table 2:** The protein levels of adipogenesis and browning markers in the tissues and serums of mice in the prevention and recovery groups (pg/mL) ^1,2,3^.

			UCP1	PGC1α	PPARγ
Prevention Group	Inguinal WAT	Control	2366.1 ± 616.56	595.8 ± 210.68	497.7 ± 211.60
		Void	2326.9 ± 428.73	468.2 ± 119.04	633.6 ± 180.37
		Free BBR	2305.8 ± 242.60	442.9 ± 115.78	530.3 ± 144.47
		Nano-BBR	2209.9 ± 473.55	491.0 ± 197.75	507.0 ± 173.33
		*p*	0.947	0.455	0.554
	BAT	Control	2016.2 ± 417.89	500.9 ± 144.95	534.6 ± 218.63
		Void	2185.7 ± 338.18	493.9 ± 94.76	504.5 ± 140.95
		Free BBR	1839.4 ± 578.98	487.0 ± 67.46	453.6 ± 13.31
		Nano-BBR	1840.4 ± 588.04	486.6 ± 85.77	480.2 ± 134.07
		*p*	0.581	0.995	0.830
	Serum	Control	6912.1 ± 3599.91	555.7 ± 335.61	2217.3 ± 1189.36
		Void	8331.5 ± 3253.07	771.2 ± 494.86	2637.8 ± 1393.68
		Free BBR	6544.4 ± 2538.78	527.5 ± 348.87	1958.8 ± 879.39
		Nano-BBR	7358.5 ± 3657.19	637.3 ± 439.18	2628.8 ± 1353.45
		*p*	0.821	0.753	0.757
Recovery Group	Inguinal WAT	Control	2589.7 ± 697.04	630.4 ± 166.41 ^a^	605.3 ± 249.78
		Void	2636.2 ± 655.39	548.3 ± 101.51 ^a^	570.9 ± 126.82
		Free BBR	2464.9 ± 395.70	390.8 ± 99.00 ^b^	500.6 ± 99.31
		Nano-BBR	2266.4 ± 401.76	513.3 ± 122.45	545.8 ± 273.23
		*p*	0.666	0.027	0.835
	BAT	Control	1883.8 ± 478.19	457.7 ± 104.45	469.6 ± 172.57 ^a^
		Void	2185.8 ± 693.20	528.5 ± 171.23	714.0 ± 320.62 ^b^
		Free BBR	2070.8 ± 744.33	496.0 ± 102.53	571.1 ± 131.98 ^a^
		Nano-BBR	2123.5 ± 536.60	476.9 ± 134.24	404.6 ± 77.88
		*p*	0.852	0.812	0.067
	Serum	Control	6407.1 ± 2834.57 ^a^	496.8 ± 387.09 ^a^	1966.0 ± 1062.07
		Void	10,984.4 ± 5607.07 ^b^	1111.9 ± 699.75 ^b^	2930.2 ± 1378.26 ^a^
		Free BBR	6028.4 ± 2485.50 ^a^	413.5 ± 263.18 ^a^	1655.9 ± 690.26 ^b^
		Nano-BBR	7607.01 ± 2890.29	740.8 ± 500.99	2220.0 ± 896.72
		*p*	0.113	0.093	0.211

^1^ Data were analyzed by one-way ANOVA. ^2^ Data are presented as mean ± SD. ^3^ Means within a column without a common letter differ, *p* < 0.05. Abbreviations: BAT, brown adipose tissue; BBR, berberine; PGC1α, PPARγ-co-activator-1α; PPARγ, peroxisome proliferator-activated receptor-γ; UCP1, uncoupling protein 1; WAT, white adipose tissue.

## Data Availability

The data that support the findings of this study are available from the corresponding author, Z.G., upon reasonable request.

## Supplementary Materials

The following supporting information can be downloaded at: https://www.mdpi.com/article/10.3390/medicina61101738/s1, Figure S1: A scanning electron microscope (SEM) (A) and final product (B) images of nano-berberine; Table S1: Primer sequences of target genes; Table S2: The physical stability of nano-berberine under different temperature and dark/light conditions; Table S3: The chemical stability of free and nano-berberine under different temperature and dark/light conditions (mcg/mL); Table S4: Hourly and accumulative in vitro release profiles for free and nano-berberine.

## Abbreviations

AMPK/SIRT1 = Adenosine monophosphate-activated protein kinase/Sirtuin 1, BAT = Brown adipose tissue, BBR = Berberine, C/EBP-β = CCAAT/enhancer-binding protein beta, CIDEA = Cell death-inducing DNA fragmentation factor-like effector A, EE = Encapsulation efficiency, ELISA = enzyme-linked immunosorbent assay, FABP4 = Fatty acid-binding protein 4, HUDHAM = Hacettepe University Laboratory Animals Research and Application Centre, LC = Loading capacity, HPLC = High-performance liquid chromatography, PCR = Polymerase chain reaction, PET/CT = Positron emission tomography combined with X-ray computed tomography, PGC1α = PPARγ-co-activator-1α, PI = Polydispersity index, PPARγ = Peroxisome proliferator-activated receptor-γ, PRDM16 = PR domain containing 16, RNA = Ribonucleic acid, SEM = Scanning Electron Microscope, soy PC = L-α-Phosphatidylcholine, UCP1 = Uncoupling protein 1, WAT = White adipose tissue.
