# Supplementary material for: Nano-Encapsulated Berberine Is a Potential Therapeutic Agent for Adipose Tissue Browning in C57BL/6J Mice
Source: Medicina (Kaunas). 2025 Sep 24;61(10):1738. doi: 10.3390/medicina61101738 (PMC12566392; doi:10.3390/medicina61101738)
Supplement: Supplementary file 1 [file medicina-61-01738-s001.zip › medicina-3822396-supplementary.pdf]

Nano-Encapsulated Berberine is a Potential Therapeutic Agent on Adipose Tissue Browning in C57BL/6J Mice

Aslıhan Alpaslan[1], Kübra Uçar Baş[1], Elif Didem Örs Demet[2], Dilem Tuğal Aslan[1], Tuba Reçber[3], Süleyman Can Öztürk[4], Tugba Gulsun[5], Mustafa Çelebier[3], Zeynep Göktaş[1*]

Methodology Supplement

Preparation of treatments

Nano-BBR, free BBR, void, or PBS were prepared according to the groups. Stock solutions were prepared by dissolving BBR 15 mg/mL, soy PC 200 mg/mL, and cholesterol 50 mg/mL in chloroform for nano-BBR and void. For nano-BBR, 200 µL was taken from the BBR stock, 250 µL from the soy PC stock, and 200 µL from the cholesterol stock, while for void, 250 µL was taken from the soy PC stock and 200 µL from the cholesterol stock. The mixtures were vortexed for 1 minute. Samples were evaporated under nitrogen gas until chloroform was completely dried. For nano-BBR, 1 mL of PBS and for void, 800 µL of PBS were added and vortexed for 1 minute. To reduce the particle size, nano-BBR was sonicated in ice water for 30 minutes and void for 10 minutes. To remove insoluble substances, both treatments were centrifuged at 5000 rpm for 7 minutes. Supernatants were passed through a 13 mm-0.22 µm PVDF syringe filter 5 times and then through a sterile 25 mm-0.22 µm-PTFE filter once. For free BBR, 1 mg of BBR was dissolved in 2689.6 µL of DMSO and vortexed for 1 minute to obtain a stock solution at a concentration of 1000 µM. The stock solution was diluted to the desired concentration with PBS. A preliminary study was conducted to determine the treatment doses, and the effect of BBR on browning markers was observed in 3T3-L1 adipocytes. As a result of the study, the application of free and nano-BBR at a dose of 10 µM was deemed

appropriate. Therefore, before each injection of the prepared treatments, the concentration was measured by HPLC, and the treatments were diluted to 10 μM with PBS.

Supplementary Figure and Tables

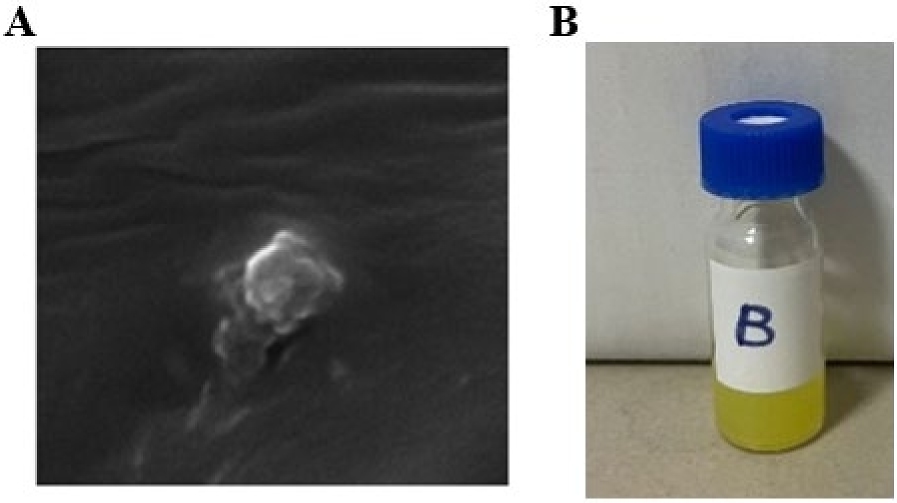

Figure S1. A scanning electron microscope (SEM) (A) and final product (B) images of nano-berberine

Table S1. Primer sequences of target genes

Gene name	Primer sequences	
PPARγ	Forward	GTACTGTCGGTTTCAGAAGTGCC
	Reverse	ATCTCCGCCAACAGCTTCTCCT
C/EBPβ	Forward	GGTTTCGGGACTTGATGCA
	Reverse	CAACAACCCCGCAGGAAC
PGC1-α	Forward	GAATCAAGCCACTACAGACACCG
	Reverse	CATCCCTCTTGAGCCTTTCGTG
CIDEA	Forward	AGAAGGTCCTACTGACCCCC
	Reverse	ACCCGGTGTCCATTTCTGTC
FABP4	Forward	TGAAATCACCGCAGACGACAGG
	Reverse	GCTTGTCACCATCTCGTTTTCTC
UCP-1	Forward	CCTGCCTCTCTCGGAAACAA
	Reverse	GTAGCGGGGTTTGATCCCAT
PRDM16	Forward	GATGGGAGATGCTGACGGAT
	Reverse	TGATCTGACACATGGCGAGG

Table S2. The physical stability of nano-berberine under different temperature and dark/light conditions

	**Nanoparticle Size**						**Polydispersity Index**						**Zeta Potential**					
	**4ºC**		**22ºC**		**37ºC**		**4ºC**		**22ºC**		**37ºC**		**4ºC**		**22ºC**		**37ºC**	
	**Light**	**Dark**	**Light**	**Dark**	**Light**	**Dark**	**Light**	**Dark**	**Light**	**Dark**	**Light**	**Dark**	**Light**	**Dark**	**Light**	**Dark**	**Light**	**Dark**
**2nd hour**	238.57	218.50	249.40	268.07	244.00	243.67	0.32	0.32	0.31	0.30	0.35	0.27	-28.80	-28.93	-28.50	-29.13	-27.57	-26.70
**4th hour**	274.30	223.73	272.13	272.37	239.33	272.87	0.42	0.33	0.32	0.25	0.30	0.28	-27.57	-27.93	-29.50	-28.47	-27.30	-27.80
**6th hour**	261.63	226.17	294.07	281.60	291.47	306.77	0.32	0.31	0.34	0.33	0.35	0.34	-27.90	-28.17	-29.53	-28.43	-28.53	-27.33
**8th hour**	222.03	250.93	260.80	263.47	288.53	305.00	0.36	0.33	0.38	0.32	0.37	0.31	-27.77	-27.50	-28.10	-30.17	-29.67	-29.93
**10th hour**	251.40	175.67	289.67	299.23	315.70	366.27	0.34	0.29	0.30	0.29	0.28	0.35	-29.23	-30.23	-29.77	-28.60	-28.10	-28.67
**2nd day**	285.00	232.83	253.40	287.33	331.97	489.23	0.40	0.35	0.34	0.34	0.37	0.45	-26.93	-27.90	-23.27	-28.03	-29.70	-29.73
**3rd day**	272.30	241.13	465.53	392.23	489.07	809.27	0.37	0.37	0.46	0.41	0.43	0.56	-27.47	-28.33	-28.10	-29.07	-24.63	-30.87
**4th day**	251.57	268.37	348.57	461.40	494.73	923.00	0.33	0.33	0.36	0.46	0.40	0.51	-28.67	-28.90	-30.57	-27.73	-30.60	-30.43
**5th day**	237.53	236.87	372.73	680.70	478.83	1.225.33	0.35	0.32	0.37	0.51	0.46	0.71	-27.77	-28.33	-29.57	-29.47	-29.77	-31.50
**10th day**	262.83	242.17	467.57	797.17	794.40	1.856.33	0.36	0.31	0.48	0.55	0.46	0.92	-30.20	-30.03	-32.50	-31.27	-33.00	-31.70

Table S3. The chemical stability of free and nano-berberine under different temperature and dark/light conditions (mcg/mL)

Time (day)	Free Berberine						Nano-Berberine					
	4ºC		22ºC		37ºC		4ºC		22ºC		37ºC	
	Light	Dark	Light	Dark	Light	Dark	Light	Dark	Light	Dark	Light	Dark
**Baseline**	4978.90						115.84					
**1st**	2887.47	2848.20	3040.47	2954.27	2842.45	3022.75	189.65	89.51	92.08	156.19	86.05	116.39
**2nd**	2846.66	2781.40	2740.78	2566.92	2320.49	1856.47	159.64	72.61	93.68	149.41	86.95	114.09
**3rd**	2748.62	2844.87	2452.58	2467.72	2570.49	2307.20	153.21	68.05	72.98	116.23	81.96	115.20
**4th**	3279.12	2757.44	2180.50	3035.56	2585.82	2797.37	152.77	73.40	90.48	142.43	91.88	103.25
**5th**	2703.27	3603.17	2335.42	2669.75	2938.40	3632.86	142.88	78.61	93.82	146.43	90.29	123.18
**10th**	3373.60	2527.55	2285.85	2368.21	3798.62	1537.33	120.06	64.07	83.22	77.66	81.11	119.23

Table S4. Hourly and accumulative in vitro release profiles for free and nano-berberine

	**Free Berberine**		**Nano-Berberine**	
**Time (h)**	**Released mass (mcg/mL)**	**Accumulative Release (%)**	**Released mass (mcg/mL)**	**Accumulative Release (%)**
**1st**	2217.75	15.58	143.19	7.23
**2nd**	3153.12	22.15	146.25	7.39
**4th**	1981.18	13.91	244.24	12.34
**6th**	1347.05	9.46	268.89	13.59
**8th**	1280.98	9.00	281.97	14.25
**10th**	1149.14	8.07	291.58	14.73
**12th**	1574.78	11.06	292.30	14.77
**24th**	1533.94	10.77	310.83	15.70
